# Detection Rates of Prostate Cancer Across Prostatic Zones Using Freehand Single-Access Transperineal Fusion Biopsies

**DOI:** 10.3390/cancers17132206

**Published:** 2025-06-30

**Authors:** Filippo Carletti, Giuseppe Reitano, Eleonora Martina Toffoletto, Arianna Tumminello, Elisa Tonet, Giovanni Basso, Martina Bruniera, Anna Cacco, Elena Rebaudengo, Giorgio Saggionetto, Giovanni Betto, Giacomo Novara, Fabrizio Dal Moro, Fabio Zattoni

**Affiliations:** Department Surgery, Oncology and Gastroenterology, Urology Clinic, University of Padua, 35126 Padua, Italy

**Keywords:** prostate cancer, transperineal, freehand, single access, detection rate, prostate zone, anterior, apex

## Abstract

**Background/Objectives**: It remains unclear whether certain areas of the prostate are more difficult to accurately sample using MRI/US-fusion-guided freehand single-access transperineal prostate biopsy (FSA-TP). The aim of this study was to evaluate the detection rates of clinically significant (cs) and clinically insignificant (ci) prostate cancer (PCa) in each prostate zone during FSA-TP MRI-target biopsies (MRI-TBs) and systematic biopsies (SB). **Methods**: This monocentric observational study included a cohort of 277 patients with no prior history of PCa who underwent 3 MRI-TB cores and 14 SB cores with an FSA-TP from January to December 2023. The intraclass correlation coefficient (ICC) was assessed to evaluate the correlation between the Prostate Imaging–Reporting and Data System (PI-RADS) of the index lesion and the International Society of Urological Pathology (ISUP) grade stratified according to prostate zone and region of index lesion at MRI. Multivariate logistic regression analysis was conducted to identify factors associated with PCa and csPCa in patients with discordant results between MRI-TB and SB. **Results**: FSA-TP-MRI-TB demonstrated higher detection rates of both ciPCa and csPCa in the anterior, apical, and intermediate zones when each of the three MRI-TB cores was analysed separately (*p* < 0.01). However, when all MRI-TB cores were combined, no significant differences were observed in detection rates across prostate zones (apex, mid, base; *p* = 0.57) or regions (anterior vs. posterior; *p* = 0.34). Concordance between radiologic and histopathologic findings, as measured by the intraclass correlation coefficient (ICC), was similar across all zones (apex ICC: 0.33; mid ICC: 0.34; base ICC: 0.38) and regions (anterior ICC: 0.42; posterior ICC: 0.26). Univariate analysis showed that in patients with PCa detected on SB but with negative MRI-TB, older age was the only significant predictor (*p* = 0.04). Multivariate analysis revealed that patients with PCa detected on MRI-TB but with negative SB, only PSA remained a significant predictor (OR 1.2, 95% CI 1.1–1.4; *p* = 0.01). In cases with csPCa detected on MRI-TB but with negative SB, age (OR: 1.0, 95% CI 1.0–1.1; *p* = 0.02), positive digital rectal examination (OR: 2.0, 95% CI 1.1–3.8; *p* = 0.03), PI-RADS score >3 (OR: 4.5, 95% CI 1.7–12.1; *p* < 0.01), and larger lesion size (OR: 1.1, 95% CI 1.1–1.2; *p* < 0.01) were significant predictors. **Conclusions**: FSA-TP using 14 SB cores and 3 MRI-TB cores ensures comprehensive sampling of all prostate regions, including anterior and apical zones, without significant differences in detection rates between nodules across different zones. Only in a small percentage of patients was csPCa detected exclusively by SB, highlighting the small but important complementary value of combining SB and MRI-TB.

## Simple Summary

Prostate cancer is one of the most common cancers in men, and accurate diagnosis is essential to guide treatment. We explored a prostate biopsy technique called freehand single-access transperineal fusion biopsy, which samples the prostate through the perineum, in a group of 277 men with a single suspicious lesion on magnetic resonance imaging of the prostate and assessed the effectiveness of this sampling method across different prostate regions. Our findings show that this technique ensures consistent access to the entire gland, including anatomically challenging areas. We also identified predictors of cancer detection in patients with discordant findings between targeted and systematic cores. These findings support its use as a safe, precise, and efficient outpatient procedure that does not require general anesthesia.

## 1. Introduction

Prostate cancer (PCa) remains a leading cause of morbidity and mortality among men worldwide [1]. Timely and accurate detection of clinically significant prostate cancer (csPCa) is essential to guide therapeutic decision-making and improve oncologic outcomes. The growing integration of magnetic resonance imaging (MRI), molecular biomarkers, and image-guided biopsy approaches reflects a shift toward tailored medicine [2,3,4]. However, diagnostics and treatments vary considerably, highlighting the need for standardized and optimized diagnostic strategies.

Although traditional MRI ultrasound-guided transrectal biopsies (TR-MRI-TBs) have long served as the preferred diagnostic approach [5], they are limited by suboptimal sampling, particularly in the anterior zone and the basal region of the prostate [6,7,8,9], which may contribute to underdiagnosis. The freehand single-access transperineal prostate biopsy (FSA-TP) has emerged as a promising alternative [10,11,12], offering comprehensive sampling of the prostate with a lower risk of infectious complications compared to transrectal methods [13,14]. Despite these advantages, uncertainties persist regarding the sampling uniformity of the FSA-TP across different prostatic zones, specifically, whether the apex, mid-gland, base, or anterior versus posterior regions present inherent challenges to accurate sample acquisition [15]. The objective of this study was to compare the detection rates of PCa and csPCa in various prostate zones using FSA-TP MRI-targeted biopsies (MRI-TBs) versus systematic biopsies (SBs). In addition, we aimed to assess the concordance between radiologic and histopathologic assessments and to identify clinical and imaging parameters that predict the presence of csPCa.

## 2. Materials and Methods

### 2.1. Patient Selection and Data Abstraction

This retrospective, single-center study included 277 biopsy-naïve patients who underwent FSA-TP between January and December 2023 at our department. Eligible patients presented with a prostate-specific antigen (PSA) level greater than 3 ng/mL [16] or a positive digital rectal examination (DRE) and with one Prostate Imaging–Reporting and Data System (PI-RADS) v.2.1 [17] score ≥ 3 lesion at multiparametric magnetic resonance imaging (mpMRI) prior to biopsy. Patients were excluded in case of prior prostate biopsy, absence of a PI-RADS ≥ 3 lesion on multiparametric MRI, incomplete or no available imaging, previous diagnosed prostate cancer, or lack of consent for data usage. Clinical data were collected from the hospital’s electronic medical records. Histopathological results and radiological variables were obtained from mpMRI reports, including Prostate Imaging–Reporting and Data System (PI-RADS) v.2.1 [17] scores, lesion size, and zonal location were retrieved and organized using REDCap electronic data capture tools hosted at University of Padua. All mpMRI scans performed outside the hospital were reviewed by a uroradiologist with 10 years of experience following European Society of Urogenital Radiology (ESUR) and European Association of Urology Section of Urologic Imaging (ESUI) guidelines [18].

### 2.2. Biopsy Technique

All FSA-TPs were performed in an outpatient setting, under local anesthesia, with patients positioned in the lithotomy position. After skin disinfection, a local anesthetic was infiltrated 1.5 cm above the anus, followed by deeper infiltration into the periprostatic space using a spinal needle. A single access with a 14G introducer needle was inserted through the anesthetized tract to facilitate the repeated passage of biopsy needles. MRI-TBs were conducted by an experienced urologist with >100 cases using MRI/US-fusion guidance to sample 3 cores from the suspicious lesion identified on mpMRI, and fusion imaging was performed using a Canon Aplio ultrasound system. SBs were performed according to a standardized 14-core template (Supplementary Figure S1), sampling peripheral and transitional zones. Histopathological analysis was conducted by experienced uropathologists with more than 10 years’ experience. For malignant lesions, grading was performed using the International Society of Urological Pathology (ISUP) system. Clinically insignificant prostate cancer (ciPCa) was defined as ISUP grade = 1, and csPCa was defined as ISUP grade ≥ 2.

### 2.3. Endpoints

Primary endpoints were the detection rates of PCa, ciPCa, and csPCa for SB, MRI-TB, and combined SB + MRI-TB according to zonal location (apex, mid-gland, base) and region (anterior, posterior). Secondary endpoints included the concordance between PI-RADS scores and biopsy ISUP grades and the correlation between zonal location and biopsy outcomes.

### 2.4. Statistical Analysis

Categorical variables were reported as frequencies, while continuous variables were expressed as medians with interquartile ranges (IQR). Differences between categorical variables were assessed using the chi-square test, while differences between continuous variables were analysed using the Student’s *t*-test or the Mann–Whitney U test, as appropriate. The intraclass correlation coefficient (ICC) with 95% confidence intervals (CI) was calculated to evaluate the concordance between PI-RADS scores and ISUP grades, as well as the relationship between zonal location and biopsy outcomes. We performed uni- and multivariate analyses to identify clinical and instrumental factors associated with the diagnosis of PCa and csPCa in patients with discordant results between MRI-TB and SB. Variables with *p* < 0.20 in univariate analysis or of clinical relevance were included in the multivariate model. All statistical analyses were conducted using SPSS version 23 (IBM, Armonk, NY, USA) and MedCalc version 20.21, with statistical significance defined as *p* < 0.05.

## 3. Results

### 3.1. Demographics

Patient characteristics and biopsy outcomes are shown in Table 1. Median age was 70 years (IQR: 64–75). A total of 14 (5.1%) patients were receiving 5α-reductase inhibitor therapy. The median PSA was 5.9 ng/mL (IQR: 4.5–8.0), with a median prostate volume of 50 cc (IQR: 38–70) and a median PSA density of 0.11 ng/mL/cc^3^ (IQR: 0.08–0.2). Median PSA free-to-total ratio was 15.5% (IQR: 12.0–21.1). Positive rectal examination was observed in 37% of patients, while 5.8% presented with a clinical stage ≥ T3 on mpMRI. The median maximum lesion diameter was 10 mm (IQR: 7–14). PI-RADS scores were distributed as follows: PI-RADS 3 in 23.5% of patients, PI-RADS 4 in 56%, and PI-RADS 5 in 20.6%. The MRI index lesion was most commonly located in the intermediate zone (46.2%), followed by apex (33.9%) and base (19.9%). When classified into anterior versus posterior regions, 68.6% were in the posterior zone and 31.4% in the anterior; in terms of zonal anatomy, 62.1% in the peripheral zone, 27.8% in the transition zone, and 10.1% in the anterior zone. MRI-TB identified PCa in 52.3%, ciPCa in 16.2%, and csPCa in 36.1% of cases. SB detected PCa in 60.3%, ciPCa in 34.7%, and csPCa in 25.6% of cases. Combined MRI-TB and SB increased detection rates to 62.8% for PCa, 39.7% for ciPCa, and 41.9% for csPCa. Discordant findings between MRI-TB and SB included 2.2% of patients with csPCa detected on SB but missed by MRI-TB, and 8.3% with csPCa detected by MRI-TB but not by SB. Additionally, 5.8% had PCa detected by SB but not MRI-TB, while 16.2% had PCa detected by MRI-TB but missed by SB.

### 3.2. Frequency Distribution of PCa and csPCa Detected by SB

**Figure 1:**
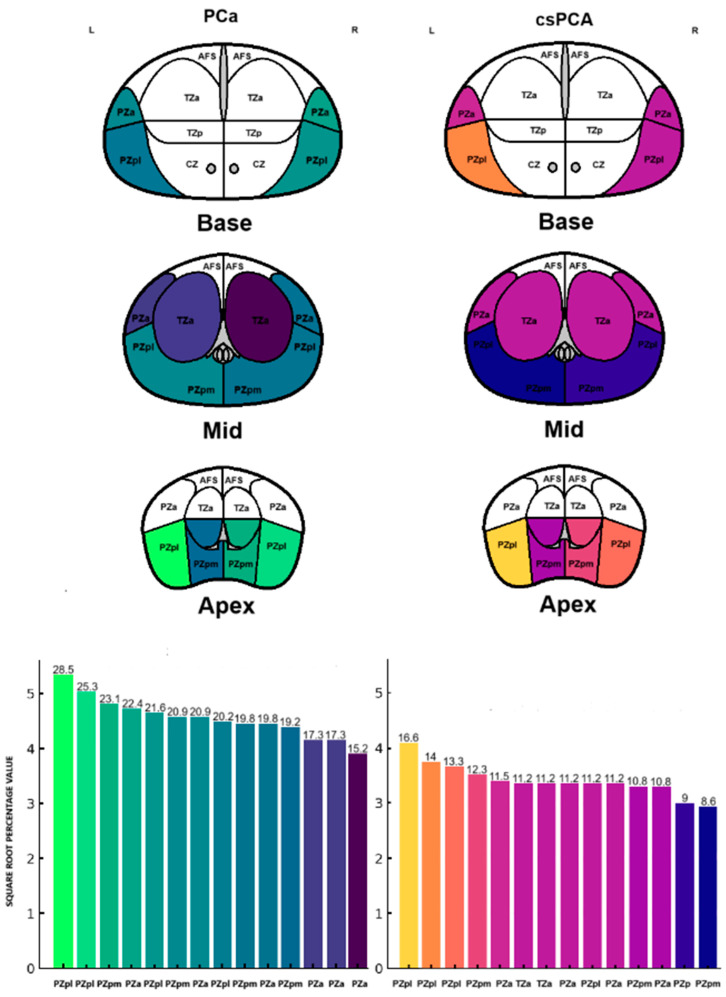
Frequency distribution of PCa and csPCa detected by SB.

Table 2 and Figure 1 summarize the frequency and percentage of PCa and csPCa detected in the 14 SB cores. The highest prevalence of PCa was observed in the right and left peripheral zone (PZpl) at the apex. In contrast, the highest frequency of csPCa was found in the right PZpl at both the apex and base. These differences in PCa and csPCA detection rates across prostate regions are not statistically significant (*p* = 0.10, *p* = 0.48).

### 3.3. Frequency Distribution of PCa and csPCa Detected by MRI-TB

**Figure 2:**
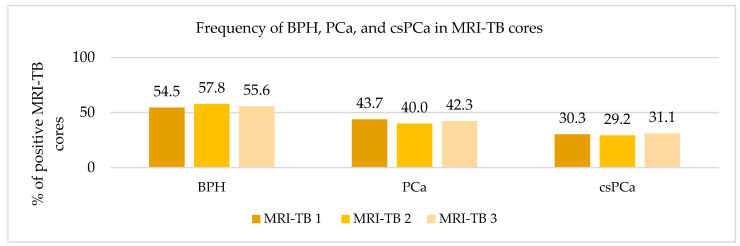
Frequency of BPH, PCa, and csPCa in MRI-TB cores.

**Figure 3:**
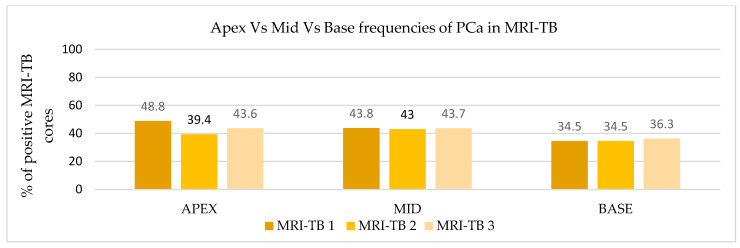
Apex vs. mid vs. base frequencies of PCa in MRI-TB.

**Figure 4:**
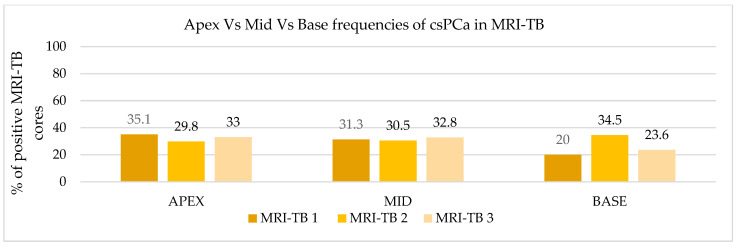
Apex vs. mid vs. base frequencies of csPCa in MRI-TB cores.

**Figure 5:**
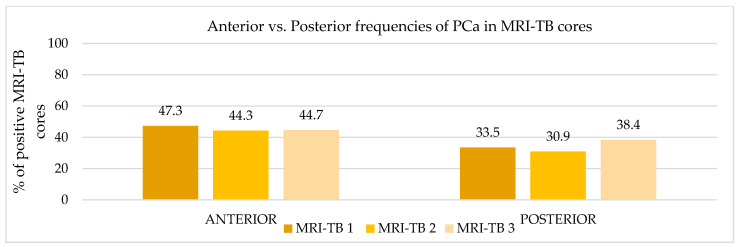
Anterior vs. posterior frequencies of PCa in MRI-TB cores.

**Figure 6:**
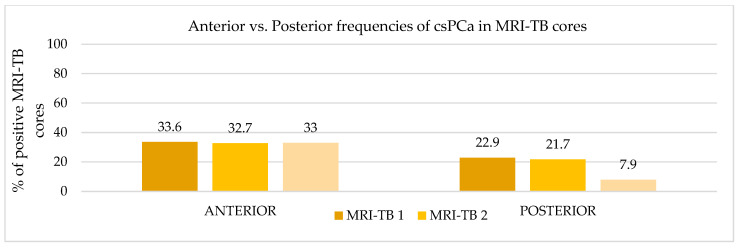
Anterior vs. Posterior frequencies of csPCa in MRI-TB cores.

Patients underwent three (IQR: 2–4) MRI-TB cores directed at the lesion identified on mpMRI. Figure 2 shows the percentage of benign tissue, PCa, and csPCa for each of the three MRI-TB cores: no significant differences in detection rates were observed among the first (MRI-TB 1), second (MRI-TB 2), or third (MRI-TB 3) targeted cores (*p* > 0.05, χ^2^ = 0.93). Analyses of MRI-TB cores were stratified by apex, mid-gland, and base for both PCa and csPCa in Figure 3 and Figure 4, respectively, as well as anterior versus posterior regions in Figure 5 and Figure 6. Table 3 shows that lesions in the apex and mid-gland showed slightly higher detection rates than those at the base, and anterior lesions had slightly higher detection rates than posterior lesions. Additionally, the frequency of PCa and csPCa was assessed for each of the three MRI-TB according to the location of the lesion within the prostate (apex, mid, and base) and the anatomical zone (anterior and posterior): the anterior region had a higher detection rate.

### 3.4. Correlation of PI-RADS, ISUP, and Lesion Location in SB vs. MRI-TB

Table 4 shows the correlation among PI-RADS, ISUP biopsy approach, and lesion location. The ICCs between the PI-RADS score and ISUP grade were 0.34 (95% CI, 0.17–0.50; *p* < 0.01) for SB, whereas MRI-TB demonstrated a higher ICC of 0.46 (95% CI, 0.32–0.57; *p* < 0.01). We further evaluated the distribution of PCa and csPCa in the anterior and posterior regions to determine how the PI-RADS score correlated with ISUP grade in each zone. The ICCs for the anterior and posterior zones were 0.42 (95% CI, 0.23–0.56; *p* < 0.01) and 0.26 (95% CI, –0.13–0.52; *p* = 0.08), respectively. Table 5 summarizes the corresponding biopsy outcomes (negative, ciPCa, and csPCa) in each region.

### 3.5. Concordance Between PI-RADS and ISUP for the Index Lesion, Stratified by Anterior vs. Posterior Regions for MRI-TB

We evaluated the distribution of PCa and csPCa for MRI-TB in the anterior and posterior regions based on the PI-RADS location. Table 6 illustrates the correlation between PI-RADS score and the corresponding ISUP grade for SB and MRI-TB stratified by region. The ICCs for the anterior and posterior zones were 0.49 (95% CI, 0.32–0.61, *p* < 0.01) and 0.44 (95% CI, 0.14–0.63, *p* < 0.01), respectively.

### 3.6. Concordance Between PI-RADS and ISUP for the Index Lesion, Stratified by Apex, Mid, and Base

Table 7 illustrates the distribution of PI-RADS and ISUP according to lesion location. In the apex, mid, and base prostatic regions, ICCs for SB was 0.29 (95% CI, −0.009–0.50; *p* = 0.03), 0.21 (95% CI, −0.05–0.42; *p* = 0.05), and 0.29 (95% CI, −0.10–0.56; *p* = 0.06), respectively, whereas MRI-TB had higher ICCs of 0.33 (95% CI, 0.06–0.53; *p* = 0.01), 0.34 (95% CI, 0.12–0.52; *p* = 0.003), and 0.38 (95% CI, 0.035–0.62; *p* = 0.017) at those same locations.

### 3.7. Clinical Predictors of PCa and csPCa in Discordant MRI-TB and SB Cases

We performed univariate and multivariate logistic regression analyses to identify clinical and instrumental factors associated with PCa and csPCa in patients with discordant results between MRI-TB and SB. Table 8 summarizes clinical predictors of patients with PCa detected on SB but not on MRI-TB. In this subgroup, older age was the only factor associated with a higher likelihood of detection of PCa by SB but not on MRI-TB. Table 9 shows predictors of PCa detection on MRI-TB but not on SB. In this subset, both age and PSA emerged as significant predictors of PCa in univariate analysis. However, in the multivariate model, only PSA remained independently associated with PCa detection (OR 1.2; 95% CI, 1.1–1.4; *p* = 0.01). Table 10 presents clinical and instrumental predictors of csPCa detected on MRI-TB and not on SB. Multivariate analysis identified age (OR 1.0: 95% CI, 1.0–1.1; *p* = 0.02), positive DRE (OR 2.0: 95% CI, 1.1–3.8; *p* = 0.03), PI-RADS score >3 (OR 4.5: 95% CI, 1.7–12.1; *p* < 0.01), and larger lesion diameter (OR 1.1: 95% CI, 1.1–1.2; *p* < 0.01) as statistically significant factors associated with the presence of csPCa in this discordant group.

## 4. Discussion

We conducted a retrospective analysis of 277 biopsy-naïve patients to evaluate the diagnostic performance of FSA-TP. Our data reinforce the growing consensus that the FSA-TP approach enables a comprehensive and reliable prostate sampling, particularly in anatomically challenging areas such as the anterior and apical zones [19].

When individual MRI-TB cores were evaluated, csPCa was more frequently detected in the anterior, apical, and mid-gland areas (*p* < 0.01). However, when the three MRI-TB cores were grouped together, detection rates were similar across zones (*p* = 0.57 for apex vs. mid vs. base; *p* = 0.34 for anterior vs. posterior), showing the reliability and consistency of the three-core strategy. Furthermore, the concordance between mpMRI findings and histopathology was higher for MRI-TB (ICC: 0.46) than for systematic biopsy (SB, ICC: 0.34), especially in the anterior zone (ICC: 0.42 vs. 0.26 posterior), with a gradual increase in concordance observed from apex to base. These findings support the precision and reliability of the FSA-TP approach across all prostatic regions. As expected, MRI-TB showed better performance in detecting csPCa compared to SB (36.1% vs. 25.6%). That said, MRI-TB missed 2.2% of csPCa cases that were only identified through SB, pointing to a small but important risk of underdiagnosis. In contrast, SB failed to detect 8.3% of csPCa cases captured by MRI-TB and more often identified clinically insignificant cancers, raising concerns about overdiagnosis. Combining MRI-TB and SB provided the highest overall detection rate for csPCa (45.5%), which is consistent with prior studies [20,21,22]. In patients with csPCa detected on MRI-TB but with negative SB, multivariate analysis showed that several factors, including older age (*p* = 0.02), a positive DRE (*p* = 0.03), PI-RADS > 3 (*p* < 0.01), and larger lesion size (*p* < 0.01), were significant predictors. These results may help guide biopsy strategy, especially when considering the omission of SB in carefully selected patients in favour of perilesional sampling strategies.

FSA-TP proved to be a reliable technique for comprehensive sampling of the prostate, including anatomically challenging regions such as the anterior and apical zones. These areas are notoriously under-sampled by the traditional transrectal (TR) approach due to its limited angulation and depth of reach, particularly in patients with large prostates or anterior lesions [6,19]. In contrast, the transperineal (TP) route provides a direct trajectory along the longitudinal axis of the gland, enhancing access to anteriorly located tumours and improving diagnostic yield in these regions. The clinical implications are relevant: in the ongoing debate between TR and TP routes [13,23], even if the detection rates have been proved similar [13], the anterior and apical regions remain hard to sample by transrectal biopsy [6,24]. Our findings support the utility of the TP approach, particularly for its ability to better sample lesions located in the anterior [25,26] and apical zones and its potential to reduce infection risk [13], allowing for office-based procedures without prophylactic antibiotics. However, given the absence of a comparative arm, no definitive conclusions can be drawn regarding its superiority over the transrectal route.

It is also to be noted that the TP approach is a well-tolerated outpatient procedure [22,27,28] with a low patient-reported pain score. Compared to more complex TP techniques, recent evidence shows that FSA-TP offers equivalent [29,30] or better [31] cancer detection rates to grid-based methods, lower complication rates [30,31], and greater efficiency in an outpatient setting [30]. The advantages of using the FSA-TP over template or double-access TP approaches are a reduced number of perineal punctures with lower procedural pain, infection risk, and shorter procedure time. It is also feasible under local anesthesia in an outpatient setting without specialized equipment such as a stepper grid or template.

As supported by recent studies, our three-core per lesion protocol appears sufficient [15]; however, there is a growing interest in whether expanding the target sampling area through additional cores or perilesional sampling, replacing SB cores, could improve the detection of csPCa. This approach is supported by recent evidence from Zattoni et al. [15], who reported that increasing the number of targeted biopsy cores up to five and including perilesional areas can significantly enhance the detection of csPCa, without substantially increasing ciPCa. This is further supported by findings from a recent multicenter study [32], which demonstrated that in patients with multiple MRI-visible lesions, the incremental value of targeting non-index lesions was minimal in csPCa detection.

In our series, all biopsies were performed in an outpatient setting, without the need for hospitalization, general anesthesia, or high-cost equipment. This not only demonstrates the procedural simplicity of the FSA-TP approach but also supports its scalability. By enabling comprehensive access to all prostate zones and reducing the risk of post-procedure complications, FSA-TP emerges as a strong candidate for becoming the standard diagnostic pathway. This is especially critical in a global context, where approximately two million prostate biopsies are performed each year, and the need to optimize resource allocation while minimizing infection risk has become increasingly critical.

Our study has several limitations: first, the absence of a comparative control group prevents direct conclusions about the relative performance of FSA-TP versus other biopsy strategies. Additionally, its retrospective single-center design, without definitive prostatectomy specimens, biopsy-related complications, or pain, may limit generalizability. Finally, although the classification of ciPCa and csPCa followed ISUP guidelines, it may vary slightly in other clinical contexts.

Future studies should validate these results in multicentred prospective settings and explore the incremental value of perilesional cores [33,34] in FSA-TP. The integration of artificial intelligence and real-time augmented reality guidance holds the potential to enhance the precision, standardization, and immersive visualization in a metaverse environment, paving the way for a new era of AI-assisted prostate cancer diagnostics.

## 5. Conclusions

MRI/US-fusion-guided freehand single-access transperineal prostate biopsy allows accurate and uniform sampling of all prostate regions, including anterior and apical areas that are often undersampled by transrectal biopsy. While MRI-TB is crucial for diagnosing csPCa, a small percentage of patients had tumors detected by SB alone, emphasizing the importance of combining MRI-TB with SB in selected patients. Its cost-effective precision, combined with outpatient feasibility, supports its role as a reliable standard for prostate cancer diagnosis.

## Figures and Tables

**Table 1:** Patient characteristics.

Variable	Whole Cohort (*n* = 277)
Median age (IQR)	70 (64–75)
Therapy with 5—ARI	14 (5.1%)
Median PSA at initial biopsy (ng/mL)	5.9 (4.5–8.0)
Median prostate volume (IQR)	50.0 (38.0–70.0)
PSAD (ng/mL/cc^3^)	0.11 (0.08–0.2)
PSA F/T	15.5 (12.0–21.1)
Positive rectal examination	98 (37.0%)
Clinical stage ≥ T3 at mpMRI	16 (5.8%)
Maximum median diameter of the lesion (mm)	10.0 (7–14)
**PI-RADS score**	
3	62 (22.4%)
4	157 (56.7%)
5	58 (20.9%)
**MRI index lesion position**	
Apex	94 (33.9%)
Intermediate	128 (46.2%)
Base	55 (19.9%)
**MRI index lesion position**	
Anterior Zone (PZa + TZa + AS)	87 (31.4%)
Posterior Zone (PZpl + CZ + PZpm + TZp)	190 (68.6%)
**MRI index lesion position**	
Periferal (PZpl + CZ + PZpm)	172 (62.1)
Transition (TZa + TZp)	77 (27.8)
Anterior (AS + PZa)	28 (10.1)
Median number of MRI-TB cores	3 (2–4)
**MRI-TB cores**	
** PCa**	145 (52.3%)
ciPCa	45 (16.2%)
csPCa	100 (36.1%)
**SB cores**	
** PCa**	167 (60.3%)
ciPCa	96 (34.7%)
csPCa	71 (25.6%)
**MRI-TB and SB cores**	
** PCa**	174 (62.8%)
ciPCa	48 (17.3%)
csPCa	123 (45.5%)
Negative MRI-TB but csPCA on SB	6 (2.2%)
CsPCa on MRI-TB but negative SB	23 (8.3%)
Negative MRI-TB but PCa on SB	16 (5.8%)
PCa on MRI-TB but negative SB	45 (16.2%)

**Table 2:** Frequency and percentage of PCa and csPCa detected in the 14 SB cores.

CORE NUMBER—ZONE SIDE	PCa *n* (%)	*p*-Value	csPCa *n* (%)	*p*-Value
**CORE 1—PZpm R apex**	53 (19.2)	0.10	30 (10.8)	0.48
**CORE 2—PZpm L apex**	64 (23.1)		34 (12.3)	
**CORE 3—PZpl R apex**	79 (28.5)		46 (16.6)	
**CORE 4—PZpl L apex**	70 (25.3)		38 (13.3)	
**CORE 5—PZpm R mid**	58 (20.9)		33 (11.9)	
**CORE 6—PZpm L mid**	55 (19.8)		24 (8.6)	
**CORE 7—PZa R mid**	48 (17.3)		25 (9)	
**CORE 8—PZa L mid**	55 (19.8)		30 (10.8)	
**CORE 9—PZpl R base**	56 (20.2)		39 (14)	
**CORE 10—PZpl L base**	60 (21.6)		31 (11.2)	
**CORE 11—PZa R base**	58 (20.9)		32 (11.5)	
**CORE 12—PZa L base**	62 (22.4)		31 (11.2)	
**CORE 13—TZa R mid**	48 (17.3)		31 (11.2)	
**CORE 14—TZa L mid**	42 (15.2)		31 (11.2)	

**Table 3:** Frequencies and zonal distribution of PCa and csPCa detected by MRI-TB cores.

		MRI-TB 1		MRI-TB 2		MRI-TB 3	
**PCa**	**Apex**	46 (48.9)	*p*-value < 0.01	37 (39.4)	*p*-value < 0.01	41 (43,6)	*p*-value < 0.01
	**Mid**	56 (43.8)		55 (43)		56 (43.7)	
	**Base**	19 (34.5)		19 (34.5)		20 (36.3)	
**csPCa**	**Apex**	33 (35.1)	*p*-value < 0.01	28 (29.8)	*p*-value < 0.01	31 (33)	*p*-value < 0.01
	**Mid**	40 (31.3)		39 (30.5)		42 (32.8)	
	**Base**	11 (20)		14 (34.5)		13 (23.6)	
**PCa**	**Anterior**	90 (47.3)	*p*-value < 0.01	84 (44.3)	*p*-value < 0.01	84 (44.7)	*p*-value < 0.01
	**Posterior**	31 (33.5)		27 (30.9)		33 (38.4)	
**csPCa**	**Anterior**	64 (33.6)	*p*-value < 0.01	62 (32.7)	*p*-value < 0.01	62 (33)	*p*-value < 0.01
	**Posterior**	20 (22.9)		21 (21.7)		24 (7.9)	

**Table 4:** Correlation among PI-RADS score, ISUP, biopsy approach, and lesion location.

		NEGATIVE *n* (%)	ISUP 1 *n* (%)	ISUP 2 *n* (%)	ISUP 3 *n* (%)	ISUP 4 *n* (%)	ISUP 5 *n* (%)
**SB**	**PI-RADS 3**	33 (11.9)	14 (5.1)	6 (2.2)	6 (2.2)	5 (1.8)	1 (0.4)
	**PI-RADS 4**	69 (24.9)	31 (11.2)	18 (6.5)	14 (5.1)	19 (6.9)	4 (1.4)
	**PI-RADS 5**	8 (2.9)	7 (2.5)	17 (6.1)	6 (2.2)	11 (4)	8 (2.9)
**MRI-TB**	**PI-RADS 3**	39 (14.1)	17 (6.1)	6 (2.2)	3 (1.1)	0 (0.0)	0 (0.0)
	**PI-RADS 4**	85 (30.7)	21 (7.6)	21 (7.6)	16 (5.8)	8 (2.9)	4 (1.4)
	**PI-RADS 5**	8 (2.9)	7 (2.5)	18 (6.5)	9 (3.2)	10 (3.6)	5 (1.8)
**ANTERIOR ZONE**	**PI-RADS 3**	23 (12.1)	12 (6.3)	5 (2.6)	5 (2.6)	2 (1.1)	0 (0.0)
	**PI-RADS 4**	45 (23.7)	18 (9.5)	15 (7.9)	9 (4.7)	16 (8.4)	3 (1.6)
	**PI-RADS 5**	4 (2.1)	4 (2.1)	9 (4.7)	4 (2.1)	10 (5.3)	6 (3.2)
**POSTERIOR ZONE**	**PI-RADS 3**	10 (11.5)	2 (2.3)	1 (1.1)	1 (1.1)	3 (3.4)	1 (1.1)
	**PI-RADS 4**	24 (27.6)	13 (14.9)	3 (3.4)	5 (5.7)	3 (3.4)	1 (1.1)
	**PI-RADS 5**	4 (4.6)	3 (3.4)	8 (9.2)	2 (2.3)	1 (1.1)	2 (2.3)

**Table 5:** Summary of SB and MRI-TB outcomes in the anterior and posterior regions.

	Index Lesion Region	Negative (*n*, %)	*p*-Value	ciPCa (*n*, %)	*p*-Value	csPCa (*n*, %)	*p*-Value
**SB**	**Anterior**	72 (37.9)	0.39	67 (35.2)	0.82	51 (26.8)	0.28
	**Posterior**	38 (43.6)		29 (33.3)		20 (22.9)	
**MRI-TB**	**Anterior**	84 (45.6)	0.39	27 (14.6)	0.87	73 (39.7)	0.34

**Table 6:** Concordance between PI-RADS and ISUP in anterior vs. posterior MRI-TB cores.

		NO PCa *n* (%)	ISUP 1 *n* (%)	ISUP 2 *n* (%)	ISUP 3 *n* (%)	ISUP 4 *n* (%)	ISUP 5 *n* (%)
**ANTERIOR**	**PI-RADS 3**	25 (13.2)	15 (7.9)	5 (2.6)	2 (1.1)	0 (0.0)	0 (0.0)
	**PI-RADS 4**	55 (28.9)	16 (8.4)	15 (7.9)	10 (5.3)	7 (3.7)	3 (1.6)
	**PI-RADS 5**	4 (2.1)	2 (1.1)	12 (6.3)	7 (3.7)	9 (4.7)	3 (1.6)
**POSTERIOR**	**PI-RADS 3**	14 (16.1)	2 (2.3)	1 (1.1)	1 (1.1)	0 (0.0)	0 (0.0)
	**PI-RADS 4**	30 (34.5)	5 (5.7)	6 (6.9)	6 (6.9)	1 (1.1)	1 (1.1)
	**PI-RADS 5**	4 (4.6)	5 (5.7)	6 (6.9)	2 (2.3)	1 (1.1)	2 (2.3)

**Table 7:** Concordance between PI-RADS and ISUP for the index lesion, stratified by apex, mid, and base, with SB and MRI-TB.

			NEGATIVE *n* (%)	ISUP 1 *n* (%)	ISUP 2 *n* (%)	ISUP 3 *n* (%)	ISUP 4 *n* (%)	ISUP 5 *n* (%)	Negative (*n*, %)	*p*-Value (Negative)	ciPCa (*n*, %)	*p*-Value (ciPCa)	csPCa (*n*, %)	*p*-Value (csPCa)
**SB**	**APEX**	PI-RADS 3	11 (11.7)	5 (5.3)	3 (3.2)	3 (3.2)	2 (2.1)	0 (0.0)	33 (35.1)	0.27	34 (36.2)	0.26	27 (28.7)	0.80
		PI-RADS 4	20 (21.3)	13 (13.8)	5 (5.3)	4 (4.3)	8 (8.5)	1 (1.1)						
		PI-RADS 5	2 (2.1)	3 (3.2)	3 (3.2)	2 (2.1)	6 (6.4)	3 (3.2)						
	**MID**	PI-RADS 3	12 (9.4)	6 (4.7)	2 (1.6)	1 (0.8)	3 (2.3)	1 (0.8)	47 (36.7)		50 (39.0)		31 (24.2)	
		PI-RADS 4	31 (24.2)	16 (12.5)	11 (8.6)	8 (6.3)	7 (5.5)	2 (1.6)						
		PI-RADS 5	4 (3.1)	4 (3.1)	10 (7.8)	2 (1.6)	5 (3.9)	3 (2.3)						
	**BASE**	PI-RADS 3	10 (18.2)	3 (5.5)	1 (1.8)	2 (3.6)	0 (0.0)	0 (0.0)	30 (54.5)		12 (4.3)		13 (4.7)	
		PI-RADS 4	18 (32.7)	2 (3.6)	2 (3.6)	2 (3.6)	4 (7.3)	1 (1.8)						
		PI-RADS 5	2 (3.6)	0 (0.0)	4 (7.3)	2 (3.6)	0 (0.0)	2 (3.6)						
**MRI-TB**	**APEX**	PI-RADS 3	13 (13.8)	6 (6.4)	4 (4.3)	1 (1.1)	0 (0.0)	0 (0.0)	44 (47.8)	0.89	12 (13.0)	0.70	36 (39.1)	0.57
		PI-RADS 4	20 (29.8)	7 (7.4)	7 (7.4)	5 (5.3)	2 (2.1)	2 (2.1)						
		PI-RADS 5	3 (3.2)	1 (1.1)	8 (8.5)	1 (1.1)	4 (4.3)	2 (2.1)						
	**MID**	PI-RADS 3	18 (14.1)	4 (3.1)	1 (0.8)	2 (1.6)	0 (0.0)	0 (0.0)	59 (47.2)		17 (13.6)		49 (39.2)	
		PI-RADS 4	38 (29.7)	11 (8.6)	11 (8.6)	8 (6.3)	5 (3.9)	2 (1.6)						
		PI-RADS 5	3 (2.3)	5 (3.9)	8 (6.3)	7 (5.5)	4 (3.1)	1 (0.8)						
	**BASE**	PI-RADS 3	8 (14.5)	7 (12.7)	1 (1.8)	0 (0.0)	0 (0.0)	0 (0.0)	29 (53.7)		10 (18.5)		15 (27.8)	
		PI-RADS 4	19 (34.5)	3 (5.5)	3 (5.5)	3 (5.5)	1 (1.8)	0 (0.0)						
		PI-RADS 5	2 (3.6)	1 (1.8)	2 (3.6)	1 (1.8)	2 (3.6)	2 (3.6)						

**Table 8:** Univariate logistic regression for predicting negative MRI-TB but PCa on SB.

Predictor	OR	95% CI	*p*-Value
**Age**	0.9	0.9–1.0	**0.04**
**PSA**	0.4	0.9–1.1	0.4
**PSAD**	0.9	0.5–1.6	0.8
**Prostate Volume**	1.0	1.0–1.0	0.2
**Anterior vs. Posterior**	1.9	0.8–4.1	0.1
**Apex**	1 (Ref)	-	-
**Middle**	0.9	0.4–2.0	0.8
**Base**	0.6	0.5–1.2	0.1
**Positive DRE**	0.8	0.4–1.8	0.7
**PI-RADS 3 vs. >3**	0.9	0.4–2.2	0.8
**Maximum Lesion Diameter**	1.0	0.9–1.0	0.2

**Table 9:** Uni- and multivariate logistic regression for predicting PCa on MRI-TB but negative SB.

	Univariate			Multivariate		
Predictor	OR	95% CI	*p*-Value	OR	95% CI	*p*-Value
**Age**	1.1	0.9–1.1	0.3	1.1	0.9–1.3	0.2
**PSA**	1.1	1.0–1.2	**0.02**	1.2	1.1–1.4	**0.01**
**PSAD**	0.9	0.2–3.0	0.9			
**Prostate Volume**	1.0	0.9–1.0	0.7	0.9	0.9–1.1	0.5
**Anterior vs. Posterior**	1.6	0.3–7.5	0.5	1.7	0.3–9.8	0.5
**Apex**	1	-	0.8 (Ref)	1	-	0.2 (Ref)
**Middle**	0.9	0.1–14	0.80	2.7	0.2–31.2	0.4
**Base**	0.6	0.1–7.5	0.50	7.8	0.6–9.4	0.1
**Positive DRE**	0.3	0.1–2.3	0.2	0.2	0.02–2.0	0.2
**PI** **-** **RADS (3 vs. >3)**	0.7	0.1–3.8	0.7	0.9	0.8–1.1	0.8
**Maximum lesion diameter**	1	0.9–1.1	0.9	0.9	0.8–1.1	0.8

**Table 10:** Uni- and multivariate logistic regression for predicting csPCa on MRI-TB but negative SB.

	Univariate			Multivariate		
Predictor	OR	95% CI	*p*-Value	OR	95% CI	*p*-Value
**Age**	1.0	0.9–1.0	**0.2**	1.0	1.0–1.1	**0.02**
**PSA**	1.0	1.0–1.1	**0.01**	1.2	0.9–1.2	0.1
**PSAD**	1.1	0.7–1.8	0.6			
**Prostate Volume**	0.9	0.8–0.9	**<0.01**	0.9	0.9–0.9	0.5
**Anterior vs. Posterior**	0.7	0.4–1.2	0.2	0.5	0.2–0.9	0.5
**Apex**	1	-	0.3 (Ref)	1	-	0.3 (Ref)
**Middle**	1.0	0.5–1.7	1	1.3	0.6–2.7	0.5
**Base**	0.6	0.3–1.2	0.2	0.7	0.3–1.7	0.4
**Positive DRE**	0.3	0.1–2.3	**0.2**	2.0	1.1–3.8	**0.03**
**PI** **-** **RADS (3 vs. >3)**	5.0	2.2–11.1	**<0.01**	4.5	1.7–12.1	**<0.01**
**Max. Lesion Diameter**	1.1	1.1–1.2	**<0.01**	1.1	1.1–1.2	**<0.01**

## Data Availability

The are not publicly available due to restrictions imposed by the IRB and patient confidentiality agreements but are available from the corresponding author upon reasonable request and with appropriate IRB approval.

## Supplementary Materials

The following supporting information can be downloaded at https://www.mdpi.com/article/10.3390/cancers17132206/s1, Figure S1: Prostate biopsy standardized 14-core template.

## Abbreviations

The following abbreviations are used in this manuscript:

FSA-TP	Freehand Single-Access Transperineal Prostate Biopsy
MRI	Magnetic Resonance Imaging
MRI-TB	Magnetic Resonance Imaging–Targeted Biopsy
SB	Systematic Biopsy
PCa	Prostate Cancer
csPCa	Clinically Significant Prostate Cancer
ciPCa	Clinically Insignificant Prostate Cancer
ICC	Intraclass Correlation Coefficient
PI-RADS	Prostate Imaging–Reporting and Data System
ISUP	International Society of Urological Pathology
PSA	Prostate-Specific Antigen
DRE	Digital Rectal Examination
mpMRI	Multiparametric Magnetic Resonance Imaging
IQR	Interquartile Range
REDCap	Research Electronic Data Capture
ESUR	European Society of Urogenital Radiology
TP	Transperineal
TR	Transrectal
TR-MRI-TB	Transrectal MRI-Targeted Biopsy
OR	Odds Ratio
CI	Confidence Interval
AOP	Azienda Ospedaliera di Padova
