# Supplementary figures and images for: Detection Rates of Prostate Cancer Across Prostatic Zones Using Freehand Single-Access Transperineal Fusion Biopsies
Source: Cancers (Basel). 2025 Jun 30;17(13):2206. doi: 10.3390/cancers17132206 (PMC12249212; doi:10.3390/cancers17132206)

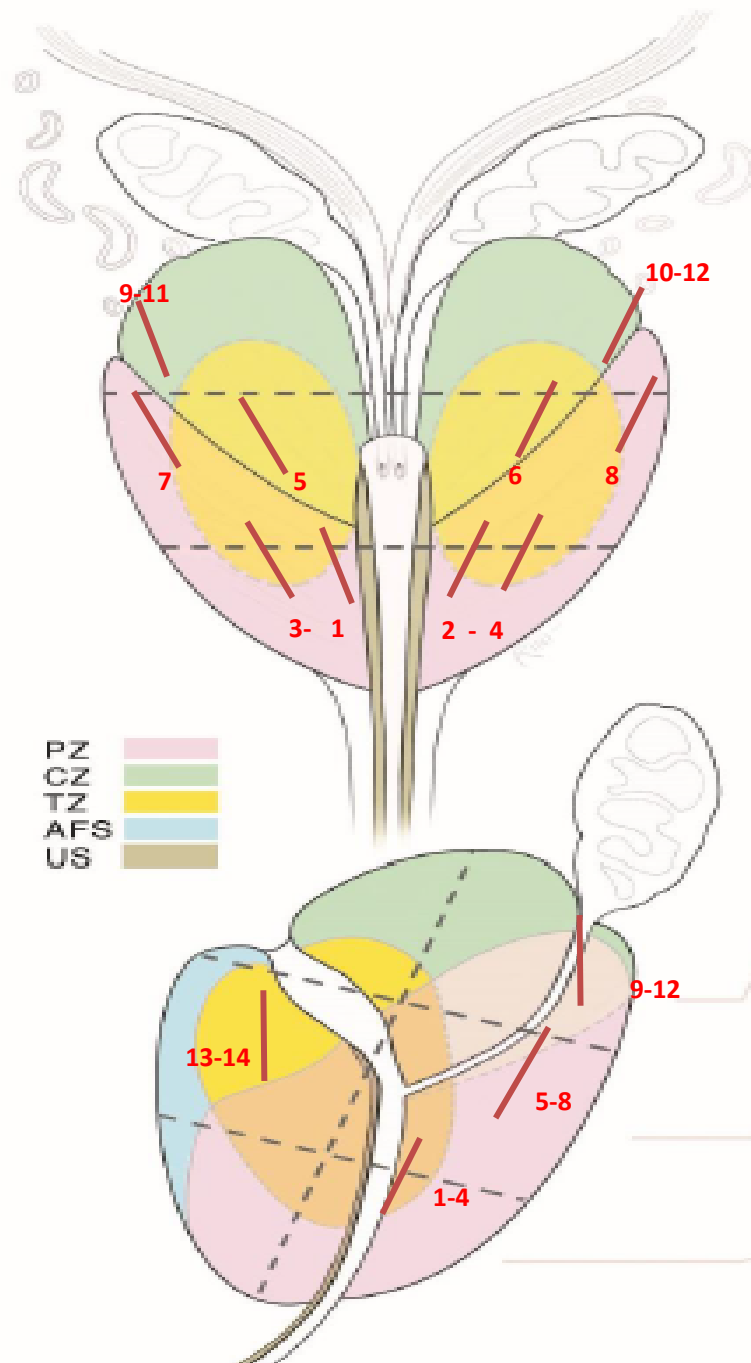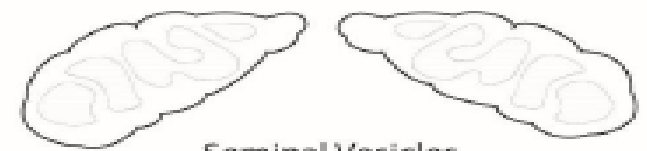

Seminal Vesicles

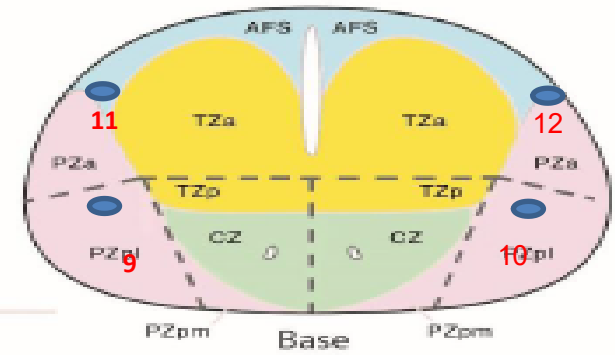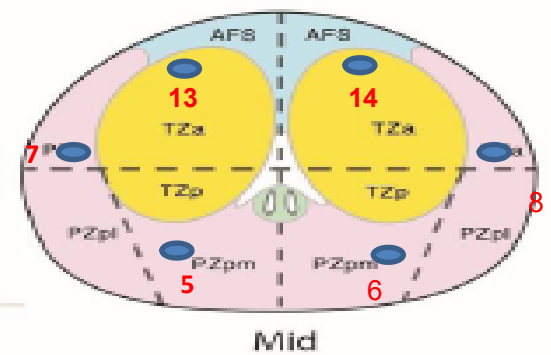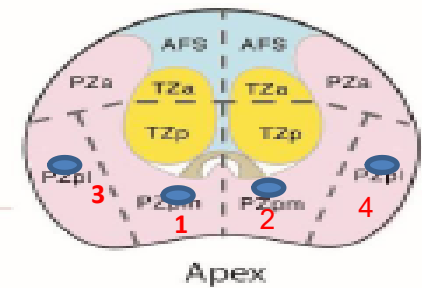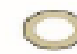

Supplement: Supplementary file 1 [file cancers-17-02206-s001.zip › Figure S1 .pdf]
